# Inhibition of Pten deficient Castration Resistant Prostate Cancer by Targeting of the SET - PP2A Signaling axis

**DOI:** 10.1038/srep15182

**Published:** 2015-11-13

**Authors:** Xiaoyong Hu, Consuelo Garcia, Ladan Fazli, Martin Gleave, Michael P. Vitek, Marilyn Jansen, Dale Christensen, David J Mulholland

**Affiliations:** 1 The 6th People’s Hospital, Shanghai Jiaotong University, Shanghai, 200233, China; 2 Icahn School of Medicine, Mount Sinai Medical Center, New York, NY, 10029, USA; 3 The Prostate Centre at Vancouver General Hospital Vancouver, British Columbia, Canada; 4 Oncotide Pharmaceuticals, Research Triangle Park, NC, 27709, USA; 5 Division of Hematology, Duke University Medical Center, Durham, NC 27710, USA

## Abstract

The PP2A signaling axis regulates multiple oncogenic drivers of castration resistant prostate cancer (CRPC). We show that targeting the endogenous PP2A regulator, SET (I2PP2A), is a viable strategy to inhibit prostate cancers that are resistant to androgen deprivation therapy. Our data is corroborated by analysis of prostate cancer patient cohorts showing significant elevation of SET transcripts. Tissue microarray analysis reveals that elevated SET expression correlates with clinical cancer grading, duration of neoadjuvant hormone therapy (NHT) and time to biochemical recurrence. Using prostate regeneration assays, we show that *in vivo* SET overexpression is sufficient to induce hyperplasia and prostatic intraepithelial neoplasia. Knockdown of SET induced significant reductions in tumorgenesis both in murine and human xenograft models. To further validate SET as a therapeutic target, we conducted *in vitro* and *in vivo* treatments using OP449 - a recently characterized PP2A-activating drug (PAD). OP449 elicits robust anti-cancer effects inhibiting growth in a panel of enzalutamide resistant prostate cancer cell lines. Using the Pten conditional deletion mouse model of prostate cancer, OP449 potently inhibited PI3K-Akt signaling and impeded CRPC progression. Collectively, our data supports a critical role for the SET-PP2A signaling axis in CRPC progression and hormone resistant disease.

Prostate cancer is driven by multiple oncogenic pathways including heightened expression of PI3K-Akt signaling which is observed in at least 90% of advanced prostate cancers^1^. Despite this, few inhibitors of PI3K-Akt signaling have had sustained clinical success. One explanation may be the rapid onset of resistance frequently achieved through acquisition of additional mutations or compensatory signaling feedback loops. An alternative approach to inhibiting PI3K-Akt dependent cancers is through the enhanced activity or expression of its key negative signaling regulators. The Protein Phosphatase 2A (PP2A) is a ubiquitously expressed serine/threonine phosphatase and key regulator of multiple oncogenic signaling pathways. Significant or partial loss of PP2A function is also a hallmark of transformation and tumorgenesis^2^,^3^. Several endogenous inhibitors of PP2A have been identified including Cellular Inhibitor of PP2A (CIP2A)^4^ and SET (also known as I2PP2A)^5^,^6^. Both oncogene proteins are direct regulators of PP2A and, thus, are predicted to regulate cancer progression in multiple tissue types.

In prostate cancer, expression and function of PP2A subunits may be lost during progression^7^,^8^ though occurring infrequently as a result of mutational inactivation^9^. Conversely, overexpression of the catalytic domain of PP2A can inhibit progression in preclinical prostate cancer models^10^. The PP2A regulator, CIP2A, can directly modulate MYC oncogenic function^11^ and is elevated in expression with respect to Gleason score in clinical prostate cancer specimens^12^. However, unlike the apparent specificity of CIP2A for inhibition of Myc^13^, the phosphoprotein SET appears to have a general PP2A inhibitory activity and, thus, may be a candidate target for treatment of PI3K/Akt driven malignancies.

Loss of the Pten tumor suppressor and resulting PI3K activation is associated both with acquisition of castration resistant prostate cancer (CRPC) and cancer progression including human cancers^1^,^14^ and mouse models of prostate cancer^15^,^16^. Given the capacity for PP2A to regulate PI3K/Akt signaling, we investigated a functional role for the SET-PP2A signaling axis in promoting prostate cancer progression. Analysis of SET RNA and protein expression in human tissue microarrays shows that SET is both increased during progression and associated with reduced time to biochemical recurrence. Gain of function analysis reveals that SET, alone, can induce pathological alterations in normal murine prostate tissue thus supporting a functional role for SET in prostate cancer progression.

Inhibition of SET through knock down studies demonstrates that enzalutamide resistant prostate cancer cells are exquisitely sensitive to reduced SET function. Using the recently described SET antagonist peptide, OP449, which has been reported to activate PP2A in multiple cancer cell types and inhibit relevant oncogenic pathways including c-Myc and Akt^13^,^17^,^18^,^19^,^20^,^21^, we found that pharmacological inactivation of SET resulted in potent growth inhibition of enzalutamide resistant prostate cancers. Collectively, our data constitute proof of principle that SET-PP2A signaling axis is a viable target for prostate cancers that may be otherwise poorly responsive to standard of care treatments.

## Results

### The PP2A inhibitor SET is overexpressed in prostate cancer

**Figure 1:**
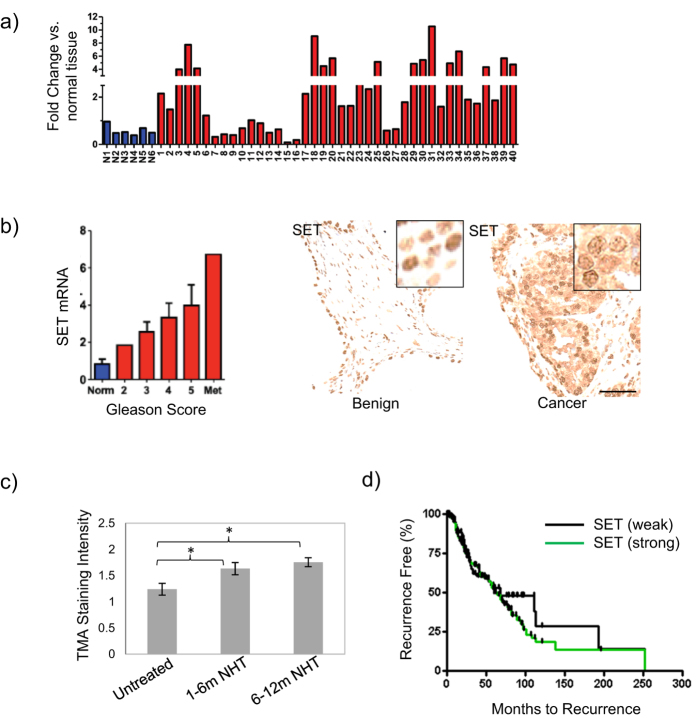
SET expression is elevated in human prostate cancer and correlates with progression. (**a**) Quantitative PCR measurement of SET in human tissue samples showing greater expression in cancer specimens (red, >2 fold, 47.5%, 19/40) compared to normal prostate samples (blue, n = 6) and (**b**)**, left,** when stratified by Gleason score (n = 8). (**b**)**, right.** Cellular localization of SET in benign and prostate cancer tissue cores. (**c)** SET expression increases in response to neoadjuvant hormone therapy (NHT) in human cohorts (patients = 88, cores = 176) (6 months NHT, p < 0.05; >12 months NHT, p < 0.05). (**d)** Elevated cytosolic SET expression is associated with reduced time to biochemical recurrence measured up to 250 m after therapy. Biochemical recurrence for patients with negative or low SET expression (0, 1 histological scoring, % recurrence = 39.1%, median recurrence = 67.8 months) in comparison to patient cancer cores with strong SET expression (2, 3 histological scoring, % recurrence = 50.6 m, median recurrence = 59.6 m).

Since PP2A expression is reduced in multiple cancers, we investigated the expression profile of the PP2A inhibitor, SET, in prostate cancer progression. Quantitative PCR (q-PCR) analysis on cDNA derived from mRNA isolated from prostate cancer samples in a commercial array obtained from Origene was used to determine that 19 of 40 (47.5%) of prostate cancer patient samples showed greater than 2 fold SET expression over normal cells (Fig. 1a). When evaluated with respect to Gleason grade SET expression was found to be highest in metastatic samples as normalized to benign prostate tissue (n = 8) (Fig. 1b).

For further validation in clinical samples, we analyzed the Taylor data set (MSKCC)^1^ and observed a significant increase in SET RNA expression both in primary tumors (p < 0.01) and metastases (p < 0.001), in comparison to normal prostate tissue (Fig. S1a). To confirm these findings at the protein level we took several approaches. First, we stained for SET using a protein array with lysates from 25 prostate cancer patients and 15 normal prostate samples spotted in triplicate on the array in 5 dilutions. Quantification of SET expression demonstrated significant elevation in cancer compared to benign tissues (p < 0.0002) (Fig. S1b, S2) - observations that were recapitulated in human prostate cancer cell lines (Fig. S1c, d). This analysis also lead us to the observation that in cancer specimens SET expression was both cytosolic and nuclear whereas it was almost exclusively nuclear in benign samples (Fig. 1b, right, Fig S2).

### SET expression is associated with androgen independent progression and biochemical recurrence

Since we observed high expression of SET mRNA in metastasis we considered whether SET could be associated with androgen independent prostate cancer progression. To do this SET protein expression was evaluated in untreated, short term neoadjuvant hormone therapy (NHT) (0–6 months treatment) and long term NHT (>12 months) clinical prostate samples (Fig. 1c, Fig. S3)^22^. In both short and long term treatment cohorts we observed a significant increase (p < 0.05) in SET expression suggesting a role in progression to castration resistance. To assign a potential role for SET expression in patient survival, we assessed a large scale TMA with known clinical patient outcomes for biochemical (PSA) recurrence over 250 months^22^. Upon pathological scoring we identified that prostate cancer cores with high SET expression (histological scoring 2–3, total 263 cores, median time to recurrence = 59.6 months, % recurrence = 50.5) had a significant decrease in the median biochemical recurrence time compared to tissue cores with low SET expression (histological scoring = 0–1, total 143 cores, median time to recurrence = 67.8 months, % recurrence = 39.1) (Fig. 1d). *Collectively, these data indicate SET is overexpressed in nearly one half of prostate cancer samples and suggest that increased SET expression correlates with progression to androgen independence and biochemical recurrence.*

### SET regulates prostate cancer progression

Previous studies have demonstrated the importance of reduced expression of PP2A for tumorgenesis and transformation^2^,^3^. Thus, we evaluated whether an associated increase in SET could be involved in prostate pathogenesis. To do this we first generated a lenti viral vector containing both SET-HA and an RFP tag (SET-FUCRW) (Fig. S4a). Upon transfection or viral infection of the SET-RW construct to 293T cells the SET-HA fusion is clearly seen migrating slightly above the endogenous SET band (lanes 2, 3; Fig. S4a, S4b) with HA expression only observed in cells containing exogenous SET-HA.

**Figure 2:**
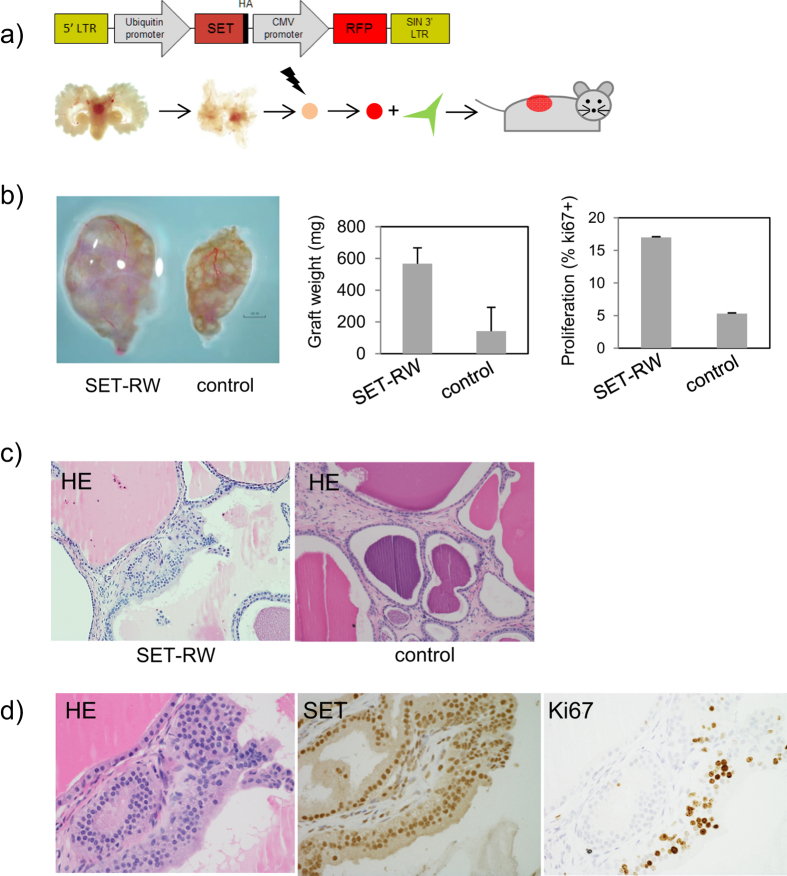
SET overexpression is sufficient to induce pathological changes in normal prostate epithelium. (**a**) Prostate tissue regenerations were constructed using normal (Wt) murine prostate epithelium infected with high titer SET lenti virus (SET-FUCRW) combined with urogenital sinus mesenchyme (UGSM). (**b**) SET lenti viral infected prostate cells generate larger grafts than control grafts (SET-FUCRW = 104.0 g, control = 38.6 g, p < 0.05) and displayed higher proliferation (SET graft = 17% Ki67, control 1%, p < 0.001). (**c**) SET lenti infected prostate cells generated grafts with pathologies encompassing hyperplasia to low grade PIN (Fig. 2c–d) (see also Fig S6a, b).

Since SET is elevated in many transformed cells, we considered whether overexpression was sufficient to alter pathology in normal (wt) prostate epithelial in culture. To do this we dissociated and plated murine prostate tissue followed by SET-FUCRW lenti viral infection and assayed for alterations in key components of the PI3K/Akt signaling pathway (Fig. S5a). Compared to control cells, we observed induced expression of SET that corresponded with significant induction of AR, P-Akt-S473, P-S6-S240 and P-4eBP1-T37/46. To test the significance of these signaling alterations on prostate cancer progression we conducted tissue recombination assays. Wild-type murine prostate epithelium were isolated to the single cell level, infected with high titer SET-FUCRW lenti virus, combined with urogenital mesenchyme (UGSM) and surgically implanted beneath the kidney capsule of nude mice or in subcutaneously in Matrigel/Media (50% vol./vol.) (Fig. 2). After 6–8 wks of incubation, grafts were harvested and analyzed for the presence of altered proliferation and pathology compared to control grafts. We observed a significant enhancement of SET-RW average graft size in comparison to controls grafts (104.0 mg vs. 38.6 mg, p < 0.05) (Fig. 2b). Correlated with this increase were alterations in pathology including enhanced proliferation, increased AR expression and formation of hyperplastic and low grade PIN (Fig. 2d, Fig. S6). To determine if increased SET expression could result in collaboration with other oncogenic events, we used PEB-1 cells, a *p53*^*−/−*^ murine cell line with minimal tumorigenic capacity^23^, infected with SET-RW virus to generate stable SET overexpressing and control PEB-1 cells. SET overexpressing cells demonstrated considerably enhanced cell viability (3 fold) by days 5 and 7 post plating (Fig. S7a). SET-PEB-1 cells also showed significantly more colony forming potential compared to PEB- control cells at a plating density of 5K per 34.6 mm (6 well) dish (p < 0.05) (Fig. S7b). *Together, these observations demonstrate that enhanced SET expression is sufficient to induce pathological alterations in Wt murine prostate epithelium and that SET may collaborate with other genetic events relevant to human prostate cancer.*

### SET inhibition leads to impaired prostate tumorgenesis

**Figure 3:**
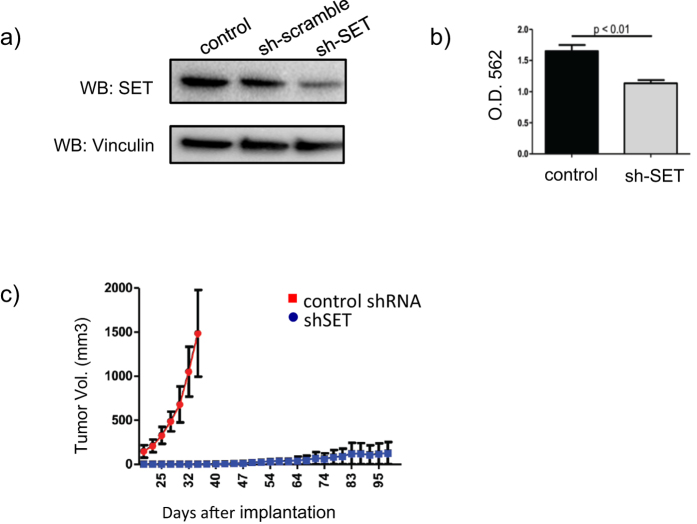
Knock down of SET impairs human prostate cancer cell growth and tumorgenesis. (**a)** Short hair pin knock down of SET in human prostate cancer (PC3) cells corresponds with (**b)** a significant inhibition of PC3 cell viability (p < 0.01) and (**c)** prevention of *in vivo* tumorgenesis measured over 100 days.

To determine if human prostate cancer cells require elevated SET expression during progression, we used lenti virus to introduced SET or control shRNAs to constitutively knock down SET in PC3 cells. Achieving approximately a 50% reduction in SET protein expression resulted in a similar decrease in cell viability, measured by alamar blue assay (p < 0.01) (Fig. 3a,b). To extend these observations *in vivo* we assessed the impact of SET knock down and the non coding shRNA control on human prostate cancer (PC3) cell tumorgenesis. To do this 2 × 10^6^ cells were injected subcutaneously to hairless SCID mice (SHO strain, Charles River) and monitored for tumor growth. Tumor volumes were evaluated at day 20 and continued until reaching 1500 mm^3^ (n = 6 per group). Strikingly, within 20 days of injection we observed tumors on 100% of the mice using PC3 cells carrying the control shRNA. In our cohort with SET knockdown, we only observed a single tumor formation at 96 days post implantation (Fig. 3c). *These data indicate that inhibition of SET expression inhibits the growth of androgen independent prostate cancer cells in vitro and significantly impairs their tumor growth in vivo.*

### SET is critical for maintenance of castrate resistant prostate cancer

**Figure 4:**
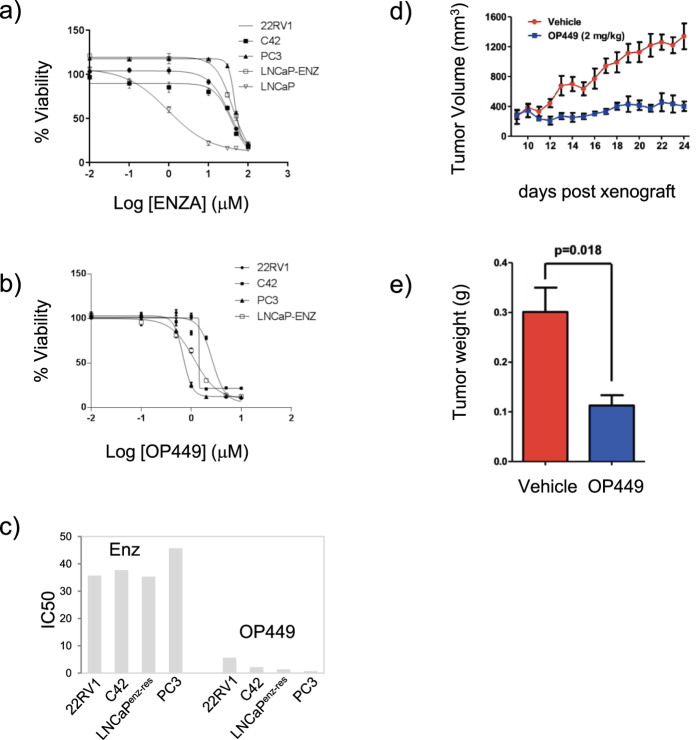
The SET antagonist, OP449, is a potent inhibitor of growth in enzalutamide resistant prostate cancer cells. (**a)** Resistant prostate cancer cells show poor response to escalating dosages of enzalutamide *in vitro* [IC_50_ (μM): 22RV1 = 35.7, C42 = 37.7, LNCaP^ENZ*−*RES ^= 35.3 and PC3 = 45.73] but are **(b,c)** highly responsive to OP449 [IC50s (μM): 22RV1 = 5.62, C42 = 2.16, LNCaP^ENZ*−*RES^, PC3 = 0.67] and **(d)** *in vivo* using PC3 cell tumorigenic assays measured by volume or **(e)** average tumor weight (n=6, treatment starting on day 9, final volume at day 24).

To investigate the therapeutic potential for targeting of SET, we tested OP449 – a small molecule inhibitor of SET^24^. For this we focused on human prostate cancer cell lines that are poorly responsive to enzalutamide. Using 22RV1, PC3 and C42 cells we evaluated growth kinetics with increasing concentrations of enzalutamide (0–100 μM) and confirmed poor responsiveness and associated high IC_50s_ when cultured in DMEM containing 5% FBS [IC_50s_ (μM): 22RV1 = 35.7, C42 = 37.7 and PC3 45.73] (Fig. 4a). Cells treated with increasing concentrations of OP449, however, showed remarkable sensitivity over 3 days of treatment with generated IC50s less than 5 μM in all lines tested [IC50s (μM): LNCaP = 1.38, 22RV1 = 5.62, C42 = 2.16, PC3 0.67] (Fig. 4b). To evaluate the *in vivo* potential of OP449 for treatment of human prostate cancer, we conducted tumorgenesis experiments using PC3 cell xenografts. Commencing treatment at day 9 post cell inoculation at 2 mg/kg, 5 days per week, we observed marked inhibition of tumorgenesis and tumor weight through 25 days post inoculation (Fig. 4c,d). *These data show that therapeutic targeting of SET using OP449 is highly efficient at preventing prostate cancer tumorgenesis.*

### OP449 is a potent inhibitor of PI3K-Akt signaling and Pten deficient castration resistant prostate cancer

**Figure 5:**
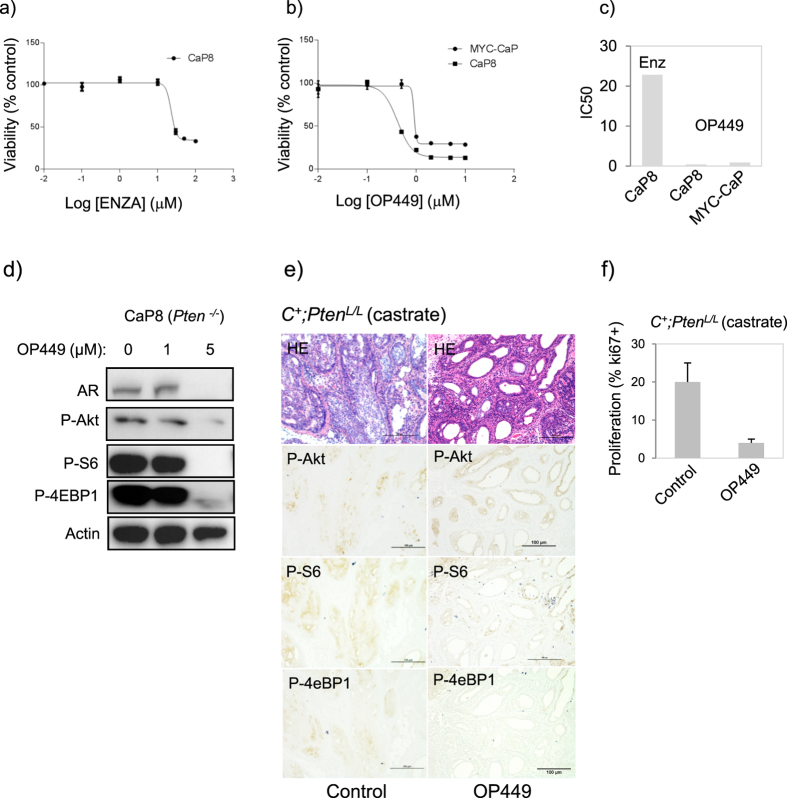
OP449 is a potent inhibitor of PI3K-Akt signaling and castration resistant prostate cancer progression. (**a**) Pten deficient murine prostate cancer cells (CaP8) show poor responsiveness to escalating doses of enzalutamide, IC_50_ = 22.84 (μM), but are (**b,c**) highly sensitive to OP449 (CaP8 IC_50 _= 0.42 μM, Myc-CaP IC_50 _= 0.9 μM). (**d**) OP449 is a potent inhibitor of PI3K/Akt signaling in Pten deficient murine prostate cancer (CaP8) cells. *In vitro* treatment (8 hours) with OP449 reduced PI3K-Akt signaling in CaP8 cells corresponding with (**e**) reduced *in vivo* PI3K-Akt signaling and CRPC progression in Pten mutant prostates (*Pb-Cre*^*+*^*;Pten*^*L/L*^, castrate) (OP449 10 mg/kg, SQ, q.a.d., commenced at 6 wks). (**f**) Proliferating (Ki67+) cells were reduced (p < 0.01) in OP449 treated mutants.

To evaluate the potential of SET inhibition for treatment of prostate cancer driven by oncogenic PI3K-Akt signaling, we tested the therapeutic effects of OP449 in treatment of an established *Pten*^*−/−*^ murine prostate cancer (CaP8) cell line^25^. Using escalating dosages we affirmed poor responsiveness to enzalutamide [IC50 (μM), CaP8 = 22.84]. However treatment with OP449 yielded IC50s of 0.42 μM (CaP8), values similar or lower IC50s than achieved with docetaxel (data not shown). Since our previous reports indicated that PI3K/Akt signaling provides a compensatory survival mechanism for reduced AR signaling^16^, we considered the effects of OP449 on PI3K/Akt signaling in CaP8 cells. Strikingly, we observed at after 8 hours of exposure to 5 μM OP449 near complete inhibition of several key signaling components (P-Akt-S473, P-S6-S240, P-4eBP1-S240, P-GSK3β-S9) was achieved. In parallel we observed significantly reduced levels of AR (Fig. 5c) (Fig S9d). Target specificity of OP449 was achieved by evaluating PP2A enzyme activity. To do this we treated CaP8 cells with escalating doses of OP449 alone and in the presence of okadaic acid (OA) – an established PP2A inhibitor (Fig. 9D). Using concentrations of 1–5 μM OP449 we observed significant increases in enzyme activity when normalized to PP2A immunoprecipitation protein levels. Co-treatment with OA (500 nM) resulted in reduced PP2A activity to near control levels. *Collectively, these data indicate that OP449 functions as a potent inhibitor of PI3K/AKT signaling operating through PP2A in CRPC cell lines.*

To investigate the importance of the SET-PP2A signaling axis for CRPC progression *in vivo* we conducted OP449 dosing using two murine models of Pten deficient prostate cancer. First, the CaP8 cell line was to generate allograft tumors. Mice were treated with either OP449 (10 mg/kg, 5 days per week) or vehicle (PBS)^13^,^19^ commencing at day 14 post-implantation and resulted in a marked separation in growth, particularly after 32 days (Fig. S8a). By day 59 post implantation control tumors reached allowable limits while growth in the OP449 treated cohort was significantly impaired as measured by tumor volume and weight (Fig S8b, c). The second *in vivo* approach applied the Pten null conditional deletion mouse model (*PB-Cre;Pten*^*L/L*^), a genetically engineered mouse model that proceeds with well defined pathological kinetics^15^. Using castrated mutant mice, dosing was carried out in mice aged 6 wks. for a total of 4 wks. with either OP449 (10 mg/kg, 5 days per week) or vehicle (PBS) according to previously conducted experiments^13^,^19^. Consistent with our previous results, control 10 wk. castrate Pten mutants demonstrated invasive pathology occurring in parallel with high expression of P-Akt, P-S6 and P-4eBP1 signaling markers (Fig. 5e). Conversely, OP449 treated mutants showed massive loss of tumor bulk and invasiveness corresponding with reduced PI3K signaling. Cell proliferation was also decreased from 18% (p < 0.05) in control mutants to 3% (p < 0.01). In many instances clusters of tunnel positive cells were observed indicating that OP449 could not only prevent tumorgenesis but also kill cancer cells (data not shown). In support of this, *in vitro* cell death assays showed that 1–5 μM OP449 treatment could induce cell death in *Pten*^*−/−*^ CaP8 cells whether measured by Annexin-FITC flow cytometry assays (Fig. S9a) or cleaved PARP (Fig. S9b). Importantly, treated mouse mutants displayed no obvious signs of toxicity measured over 4 weeks of dosing as determined by the lack of significant (>10%) weight loss (Fig. S10). *Together, these findings show that pharmacological inhibition of SET leads to inhibition of PI3K-Akt signaling and a corresponding inhibition of invasive CRPC progression.*

## Discussion

The PI3K-Akt signaling pathway is elevated in a significant portion of primary and metastatic prostate cancers^1^. Loss of the Pten tumor suppressor is both correlated with heightened PI3K-Akt signaling function and with reduced survival in prostate cancer patients^14^. Our previous studies, and those of others, demonstrated that PI3K-Akt signaling can provide compensatory survival cues for prostate epithelium with either pharmacological or genetically opposed androgen receptor signaling^16^,^26^. As a therapeutic consequence of Pten loss and PI3K-Akt mediated survival, prostate epithelium may have reduced treatment response or even resistance to anti androgen therapy^16^,^27^,^28^. The PP2A phosphatase is not only required for cell transformation^2^,^29^ but is an established regulator of Akt function. Therefore, therapeutics which are capable of modulating PP2A phosphatase function may be suitable for treatment of Pten deficient CRPC.

In this report we studied the cellular inhibitor of PP2A, SET (I2PP2A), to further understand (1) its expression patterns in prostate cancer progression, (2) its regulatory role in prostate cancer, (3) whether SET can regulate PI3K signaling and (4) if targeting SET can be exploited as a therapeutic for treatment of Pten deficient prostate cancers.

We found that SET was frequently overexpressed both at the transcript and protein level in prostate cancer cell lines and primary human cancer samples. These findings are consistent with recent observations of enhanced SET and CIP2A expression in breast^13^ and pancreatic cancers^19^. We observed that in normal prostate tissue SET expression was almost exclusively localized to the nucleus, however in clinical cancer samples was both increased in expression and localized to the cytosol. Such observations of cytoplasmic retention of SET are consistent with its ability to physically interact with PP2A and negatively regulate its activity^30^.

The above observations motivated us to question whether enhanced SET function could contribute towards prostate cancer progression. By way of tissue regeneration assays we found that exogenous SET could both enhance epithelial proliferation and induce low grade PIN mediated by an increase in P-Erk and PI3K-Akt expression. Introduction of SET into immortalized, non tumorigenic PE-B-1 cells^23^ demonstrated that enhanced SET function could also collaborate with other genetic events, such as p53 loss, to potentiate progression. Future studies will address the relative tumor initiating capacities of PP2A and its known regulators, inhibitor of PP2A (CIP2A) and SET (I2PP2A) both alone and in concert, using similar reconstitution assays.

Previous studies have exploited inhibition of SET as a means to overcome therapeutic resistance including myeloid leukemia^18^. In our clinical tissue microarray, patients who had received neoadjuvant hormone therapy (NHT) showed higher expression of SET suggesting a role in signaling pathways associated with resistance to anti androgen therapy. With this, we considered the possibility that SET inhibition and the resulting PP2A activation may be a suitable strategy to inhibit growth in castration resistant prostate cancer. To test this we used both knock down and pharmacological targeting applying the SET inhibitor OP449 as a means to activate PP2A in systems with either overt or acquired resistance to enzalutamide. Interestingly, we found that knock down or treatment with OP449 resulted in potent reductions in PI3K-Akt signaling paralleled by reduced androgen receptor expression corresponding with increased apoptosis and decreased proliferation. While the precise mechanism of how SET inhibition leads to reduced AR is unclear, interactions with P-Akt phosphorylation^31^,^32^ or restoration of PP2A activity and a subsequent alteration in ligand independent phosphorylation^7^ remain possibilities.

Our studies revealed that cell lines with either overt or acquired enzalutamide resistance were acutely responsive to OP449 treatment suggesting a potentially important role for the SET-PP2A axis in hormone resistant disease. That OP449 performed so effectively in enzalutamide resistant cells lines could be attributed to the findings that OP449 can inhibit both PI3K-Akt and AR expression. Moreover, that SET inhibition can retard progression in such genetically diverse cancers underscores the multi regulatory function of the SET-PP2A signaling axis. For example, OP449 has also shown to be effective for treatment of MYC driven solid tumors including triple negative breast cancer^13^ and pancreatic cancer harboring Ras mutations^19^. Such studies support additional benefits that may be achieved by SET inhibition in prostate cancers with MYC amplification which is frequently observed in late stage disease^1^.

In summary, our data support a role for SET inhibition as a therapeutic option for treatment of castrate resistant prostate cancer driven by PI3K-Akt activation. Given the broad spectrum of pathways regulated by PP2A it is feasible that those prostate cancers driven by MYC and AR may also benefit from therapies targeting endogenous regulators of PP2A. Future studies may consider the cooperative effects of OP449 treatment with other standard of care therapies including chemotherapy or androgen deprivation therapy.

## Methods

### Cell culture and cytotoxic assays

Human and murine prostate cancer cell lines were obtained from the American Type Culture Collection (ATCC) with the exception of C42 cells which were a gift from Dr. Leland Chung. Generation of enzalutamide resistant LNCaP cells was achieved through maintenance in 10 μM enzalutamide in 5% FBS/DMEM for three months in parallel with control treated cells. Alamar Blue assays were performed by plating 4 × 10(3) cells per well over a 48 well plate. The following day cells were treated with various concentrations of OP449 (1–10 μM). Cells were exposed to drug for 3–4 days followed by the addition of alamar blue reagent (R70717, Sigma) for 2 hours for colorimetric reading. Generation of IC50 values was achieved using Prism Software.

### Origin of cell lines and culture

CaP8 (Hong Wu, UCLA), MYC-CaP (Charles Sawyers, MSKCC), C42 (Leland Chung, Cedars Sinai), PEB-1 and PEL-1 (Lynette Wilson, NYU), LNCaP (ATCC), PC3 (ATCC), 293T (ATCC). Prostate cancer cell lines were grown in the following media conditions 5–10% (vol./vol.) FBS, 1% (vol./vol.) penicillin/streptomycin: RPMI-1640 for LNCaP, PC3 and C42. DMEM for 293T and MYC-CaP cells. PEB-1 and PEL-1 cells were grown in PrEGM (Lonza) with 1% (vol./vol.) penicillin/streptomycin and the addition of 10% only to PEL-1 cells.

### Drugs

OP449 (MW 9223 g/mol) was obtained from Oncotide Pharmaceuticals. OP449 was reconstituted in PBS as a 1 mmol/L stock and further diluted to the range of 1–10 μM for *in vitro* work or 10 mg/kg for *in vivo* studies.

### Apoptosis assay

Measurement of *in vitro* apoptosis was carried out using Annexin-FITC flo cytometry according to manufacturers instructions (Life Technology V13242).

### Western Blotting antibodies

CaP8 and Myc-CaP cell lines were cultured in the presence or absence of OP449. Following the indicated drug exposure time, cells were washed in PBS and treated with lysis buffer (Cell Signaling, #98065S or #8553S) supplemented with complete protease inhibitor PMSF (Cell Signaling). Equal amounts of protein were fractionated on 4% to 15% Tris-glycine polyacrylamide gels (#456–1086, Bio-Rad) transferred to PVDF membranes and probed with the indicated antibodies: P-Akt-S473 (#4060), P-S6-S240/244 (#5364), P-4e-BP1-T37/46 (#9459), P-GSK3β-S9 (#9336), c-Myc (D84C12,5605), Cleaved PARP (Asp214, 9544) all from Cell Signaling. AR (N-20, sc-816, Santa Cruz), HA (H6908, Sigma-Aldrich), Vinculin (V9131, Sigma-Aldrich) and β-Actin (A2103, Sigma-Aldrich), SET (Upstate, 05–421), SET (Bethyl, A302-261A) and Ki67 (Vector, VP-RMO4).

### PP2A activity assay

To measure PP2A phosphatase activity we used a commercially available assay (Upstate Biotechnology, 17–313). In brief, castration resistant CaP8 murine prostate cancer cells were treated with escalating doses of OP449 for 24 hours in DMEM (5% FBS, P/S). Cells were lysed in 20 mM imidazole HCl, 2 mM EDTA, 2 mM EGTA, pH 7.0 with 10 μg/mL each of aprotinin, leupeptin, pepstatin, 1 mM benzamidine, and 1 mM PMSF. Eighty micrograms of lysate was then immunoprecipitated with 4 μg of anti-PP2A antibody (Upstate Biotechnology, #05–421) and 40 μL of protein-A-agarose beads for 1–2 hours rotating at 4C. Beads were washed well with lysis buffer followed with the Ser/Thr assay buffer phosphatase reaction for measurment of dephosphorylation of the phosphopeptide (K-R-pT-I-R-R) through malachite green phosphate detection. The level of free phosphate was normalized to immunoprecipitated PP2Ac measured by western blot densitometry.

### Plasmids

To construct the SET-FUCRW lent viral vector, the SET cDNA was amplified from the pcDNA-SET-FLAG-HA plasmid (Addgene: 24998) with 5′ EcoR1 and 3′ Hpa1 restriction overhangs. The EcoR1-SET-Hpa1 PCR product was ligated to the –FUCRW lenti viral vector.

### SET knock down studies

Transient knockdowns were performed using siRNAs to inhibitor-2 of protein phosphatase 2A (I2PP2A/SET). Non targeting (NT) siRNA (NT siRNA) was used as a control. For the xenograft assay, cells were transfected with siRNAs two times over 2 d before transplant. Briefly, cells were transfected using Lipofectamine 2000 (Invitrogen) and stable clones were generated after selection with puromycin.

### Primary prostate cultures and regeneration assays

CD1 mouse prostate tissue propagated by mechanical dissociation followed by 16 hours of enzymatic digestion (Collagenase type 1, 1 mg/ml). Tissue was then briefly exposed to trypsin and passaged through a 21G needle, pelleted and plated in PrEGM media (Lonza). Two days later tissue was infected with SET-FUCRW lenti virus. Prostate regeneration assays were performed as previously described^16^.

### Experimental Animals

*Pb-Cre*^*+*^*;Pten*^*L/L*^ (FVB-129) mice were generated by crossing *Pten*^*L/L*^ (129, JAX) with ARR2-Pb-Cre (FVB, NCI). All mice were maintained in The Mount Sinai Medical Center, Division Laboratory of Animal Medicine. Male Nude (Nu/J, Stain 002019, Jackson Labs) or SCID (SHO hairless SCID, Charles River) mice were used for kidney capsule transplantations or subcutaneous tumor cell injections. Male CD-1 (Strain 022, Charles River) mice were used for a source of Wt prostate tissue.

### Use of mouse lines

All mice were maintained in a temperature and humidity controlled, pathogen free housing units, with light-dark cycles (10 hours light, 14 hour dark) allowing food and water distribution *ad libitum*. Breeding and experimental procedures were performed according to mouse protocols approved by the Institutional Animal Use and Care Committee (IACUC) at the Icahn School of Medicine at Mount Sinai.

### Subcutaneous tumor studies

For mouse tumorgenesis studies, 1 × 10^6^ (CaP8) or 2 × 10^5^ (Myc-CaP) cells were reconstituted in 100% Matrigel (BD) for each injection site. Once tumors were palpable, mice were randomly divided into two groups and treated via intraperitoneal injection with OP449 (10 mg/kg, 3 d/wk.) or vehicle control (PBS). Tumors were measured by a digital caliper and tumor volume was calculated by (width × length^2^)/2.

### Immunohistochemistry

Tissue staining was performed as previously described^16^,^33^. Primary antibody detection was carried out using biotinylated anti-rabbit (BA-1000) and biotinylated anti-mouse secondary antibodies (MOM Kit, BMK-2202) from Vector. DAB exposure was done using chromogen from Biogenex (HK130-5K). TUNEL assays were performed by using the *in situ* cell death detection kit from Roche (11-684-817-910).

### Real-time PCR

RNA was extracted by using Qiagen RNeasy Mini kit according to manufacturer instructions. Total RNA (3 mg in 25 ml) was reversely transcribed and produced cDNA that was used to detect the transcripts using the Rotor-Gene 3000 Real-Time PCR Detection System (Corbett Research, Sydney, NSW, Australia) with SYBR® Premix Ex Taq™. The expression level of GADPH housekeeping gene was used for normalization of SET mRNA expression level. Forward primer: 5’-CTTCAACTCTGGTCAAATAATGCA-3’, reverse primer: 5’-GAACAAAAATATAACAAACTCCGC-3’, forward primer: 5′-GAAGGTGAAGGTCGGAGTC-3′ and reverse primer: 5′-GAAGATGGTGATGGGATTTC-3′ for GADPH.

### Statistics

SD for all graphs was calculated from three independent experiments (unless otherwise stated in the figure legend) using Graph Pad Prism 5. P values were analyzed by Student t test, with a two-tailed method (P < 0.05).

## Additional Information

**How to cite this article**: Hu, X. *et al.* Inhibition of Pten deficient Castration Resistant Prostate Cancer by Targeting of the SET - PP2A Signaling axis. *Sci. Rep.* **5**, 15182; doi: 10.1038/srep15182 (2015).

## Supplementary Material

Supplementary Information
