# Supplementary material for: Inhibition of Pten deficient Castration Resistant Prostate Cancer by Targeting of the SET - PP2A Signaling axis
Source: Sci Rep. 2015 Nov 13;5:15182. doi: 10.1038/srep15182 (PMC4643319; doi:10.1038/srep15182)
Supplement: Supplementary Information [file srep15182-s1.pdf]

Scientific Reports

Supplemental Information

Inhibition of Pten deficient Castration Resistant Prostate Cancer Through Targeting of the SET - PP2A Signaling axis.

Xiaoyong Hu, Consuelo Garcia, Ladan Fazli, Martin Gleave, Michael Vitek, Marilyn Jansen, Dale Christensen and David J Mulholland

Supplemental Figures

Fig S1. Increased SET expression in metastatic prostate cancer. a) Significant increase in SET expression using the MSKCC data set[1] evaluated in primary (p<0.01) and metastatic prostate cancer (p<0.001) with respect to normal tissue. b) SET protein expression is increased in prostate cancer as measured using a protein array of human prostate samples. c) Elevated SET protein expression in prostate cancer cell lines (PrEc3, LNCaP, PC3) and d) densitometry measurements (O.D.) for SET and PP2Ac normalized to tubulin loading controls. e) Increased expression of inactivated PP2A (P-PP2A-Y307) in CRPC cancer cells *in vitro* (CaP8, 22RV1, C42) and *in vivo* (Pten mutant, $C^{+};Pten^{L/L}$) compared to normal cells.

Fig S2. SET localization in normal (a) and cancerous (b) tissue cores (bar = 100 μM).

Fig S3. Increased SET expression in patient samples treated with Neoadjuvant Hormone Therapy (NHT).

Fig S4. Schematic and expression validation of the SET lenti viral vector (SET-FUCRW). a) Human SET cDNA was amplified and sub cloned to the FUCRW lenti viral vector followed by b) validation of SET-HA expression using 293T cells either transfected (lane 2) or infected with SET-FUCRW lenti virus (lane 3).

Fig S5. SET overexpression induces signaling alterations in normal murine prostate tissue. a) Normal prostate tissue was dissociated to single cell level and cultured in PrEGM media. The following day cells were infected with SET-FUCRW lenti virus and evaluated for alterations in key drivers of prostate cancer including AR and PI3K/Akt signaling with in 24-48 hours. b) Densitometry readings for western blotting comparing Wt epithelium with and without SET overexpression each lane normalized to the loading control, vinculin.

Fig S6. Representative tissue stains from prostate regeneration experiments. a) Normal murine prostate tissue grafts and b) different pathologies observed with SET-RW lenti viral tissue recombinants.

Fig S7. SET overexpression induces (a) enhanced proliferation and (b) colony formation in immortalized $p53^{-/-}$ PEB1 prostate cells.

Fig S8. OP449 inhibits tumorgenesis in Pten deficient subcutaneous tumors. a) Mice were treated every 2 days with either 10 mg/kg OP449 or vehicle control beginning at 14 days post implantation of $Pten^{-/-}$ murine prostate cancer (CaP8) cells. Mean tumor volumes over the

course of treatment and **b)** comparisons of final tumor burden between OP449 treated and vehicle treated CaP8 tumors harvested on day 59 post implantation. **, $p < 0.001$. **c)** Representative resected tumors.

Fig S9. OP449 induces apoptosis in Pten deficient prostate cancer cells. *Pten*$^{-/-}$ murine prostate cancer (CaP8) cells were treated with treated with OP449 and evaluated for induced Annexin-FITC expression after 48 hours by (a) flow cytometry or (b) induction of cleaved PARP and p27. (c) OP449 inhibits PI3K/Akt signaling and mediates a reduction in P-GSK3β-S9 in murine (CaP8) and human CRPC (22RV1, C42) cell lines. d) Increased PP2A phosphatase activity in Pten deficient murine prostate cancer (CaP8) cells when treated with escalating doses of OP449. CaP8 cells were treated for 24 hours as control, OP449 or OP449 + okadaic acid (OA).

Fig S10. OP449 does not promote significant weight loss in mice. Line graphs show mouse individual weights before and after OP449 (10 mg/kg, 5 days/wk.) or vehicle treatment.

Fig S1

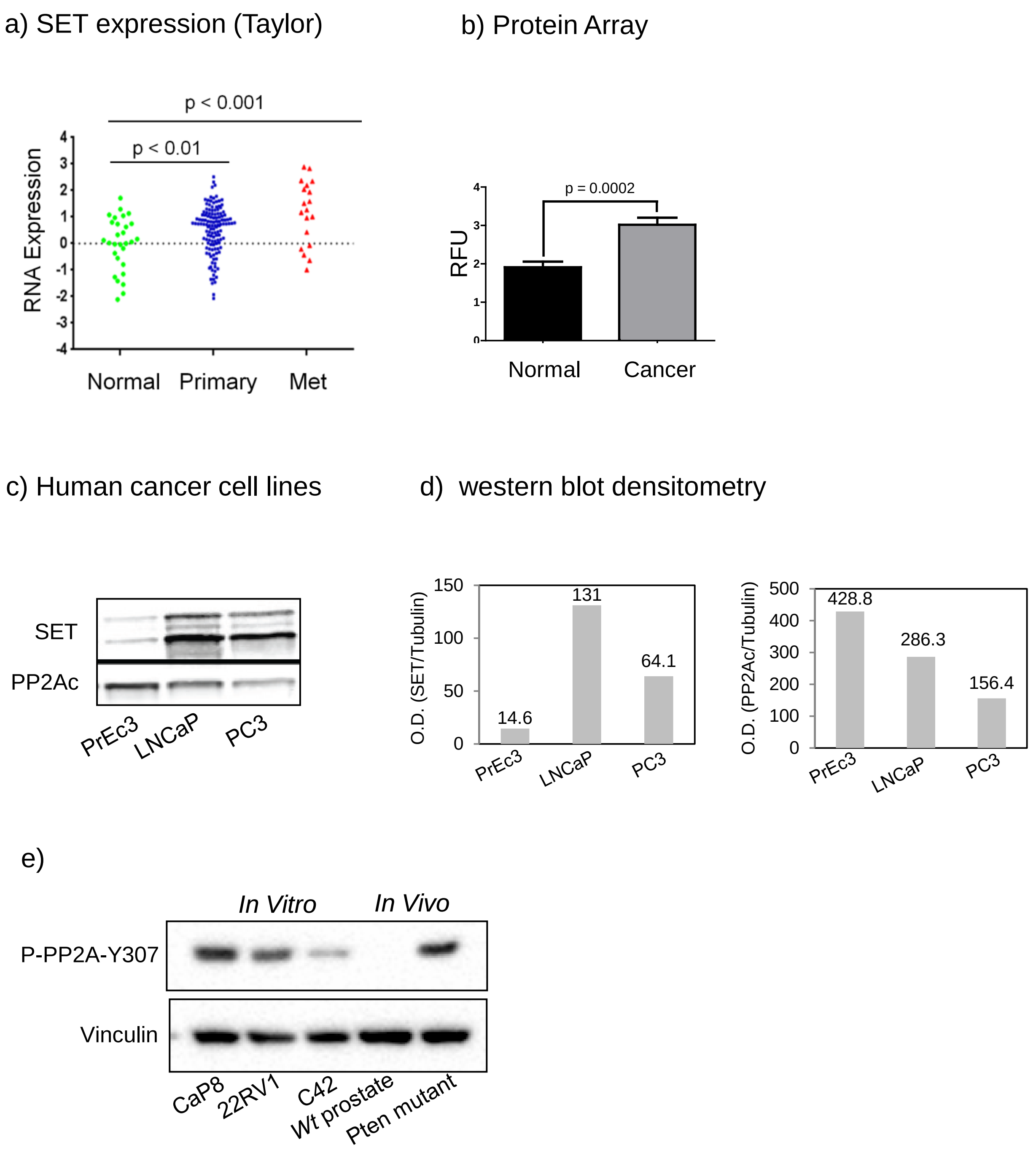

a) SET expression (Taylor)
p < 0.001
p < 0.01
RNA Expression
4
3
2
1
0
-1
-2
-3
-4
Normal
Primary
Met
b) Protein Array
p = 0.0002
RFU
4
3
2
1
0
Normal
Cancer
c) Human cancer cell lines
SET
PP2Ac
PrEc3
LNCaP
PC3
d) western blot densitometry
O.D. (SET/Tubulin)
150
100
50
0
14.6
131
64.1
PrEc3
LNCaP
PC3
O.D. (PP2Ac/Tubulin)
500
400
300
200
100
0
428.8
286.3
156.4
PrEc3
LNCaP
PC3
e)
In Vitro
In Vivo
P-PP2A-Y307
Vinculin
CaP8
22RV1
C42
Wt prostate
Pten mutant

Fig S2

a) Normal human prostate

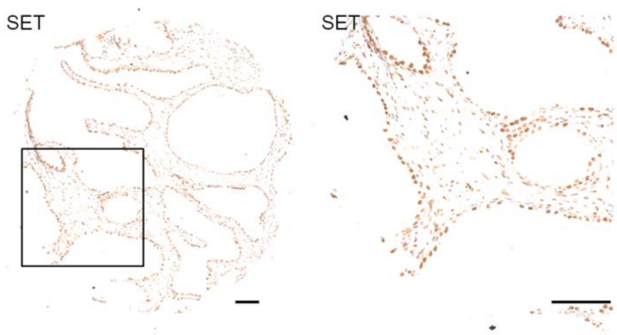

b) Human prostate cancer

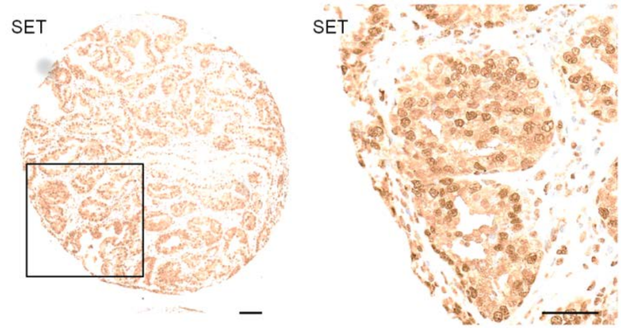

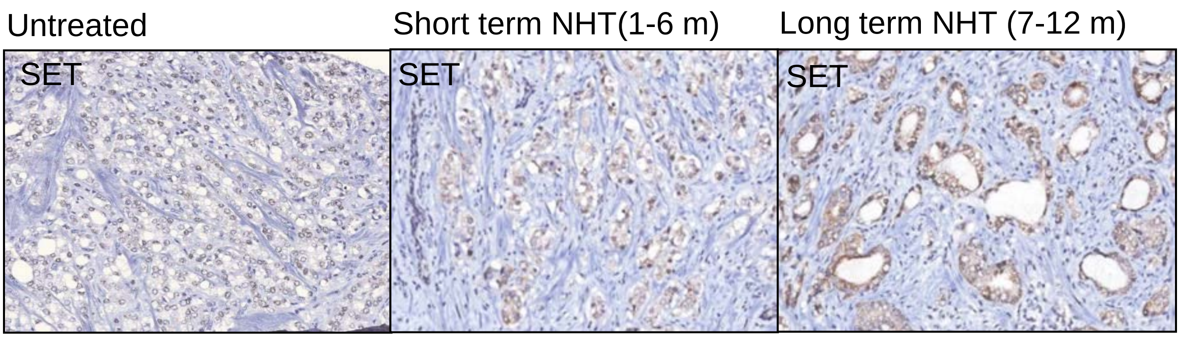
Untreated
Short term NHT(1-6 m)
Long term NHT (7-12 m)
SET
SET
SET

Fig S4

a)

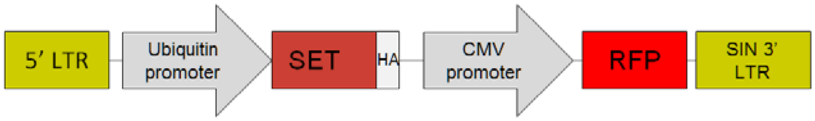

5' LTR
Ubiquitin promoter
SET
HA
CMV promoter
RFP
SIN 3' LTR

b)

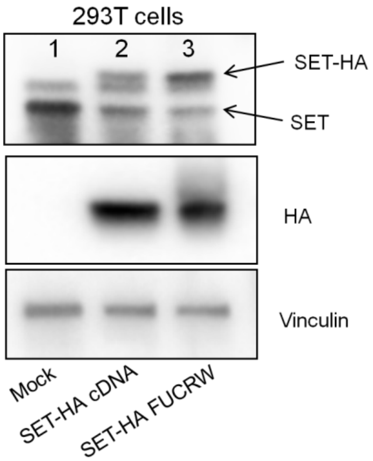

293T cells
1
2
3
SET-HA
SET
HA
Vinculin
Mock
SET-HA cDNA
SET-HA FUCRW

a)

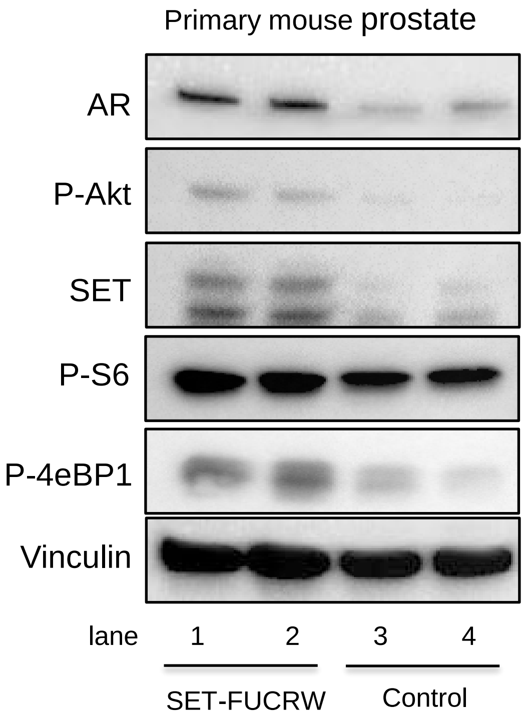
Primary mouse prostate
AR
P-Akt
SET
P-S6
P-4eBP1
Vinculin
lane
1
2
3
4
SET-FUCRW
Control

b)

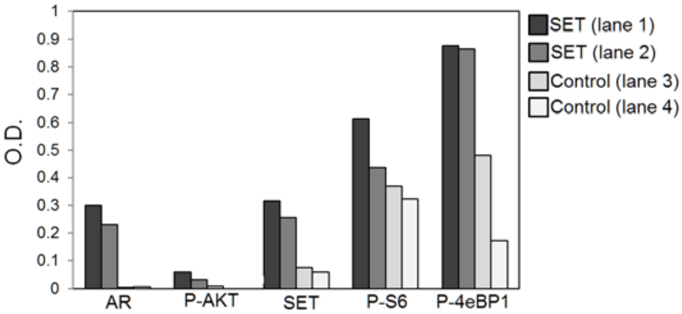
O.D.
1
0.9
0.8
0.7
0.6
0.5
0.4
0.3
0.2
0.1
0
AR
P-AKT
SET
P-S6
P-4eBP1
SET (lane 1)
SET (lane 2)
Control (lane 3)
Control (lane 4)

Fig S6

a) Control Graft

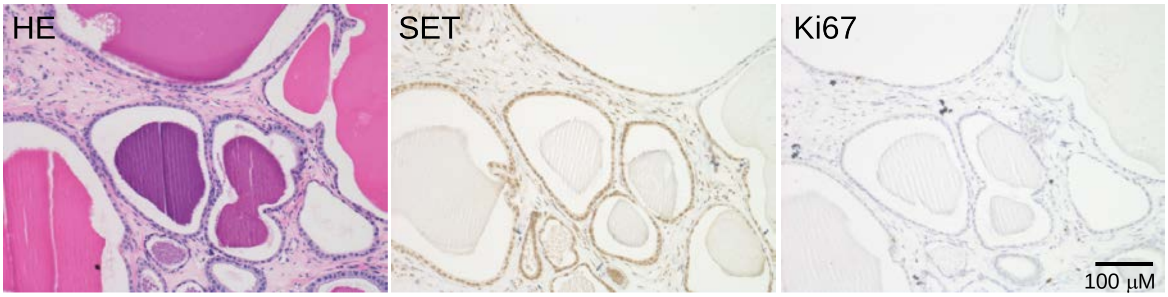

b) SET-RW Graft

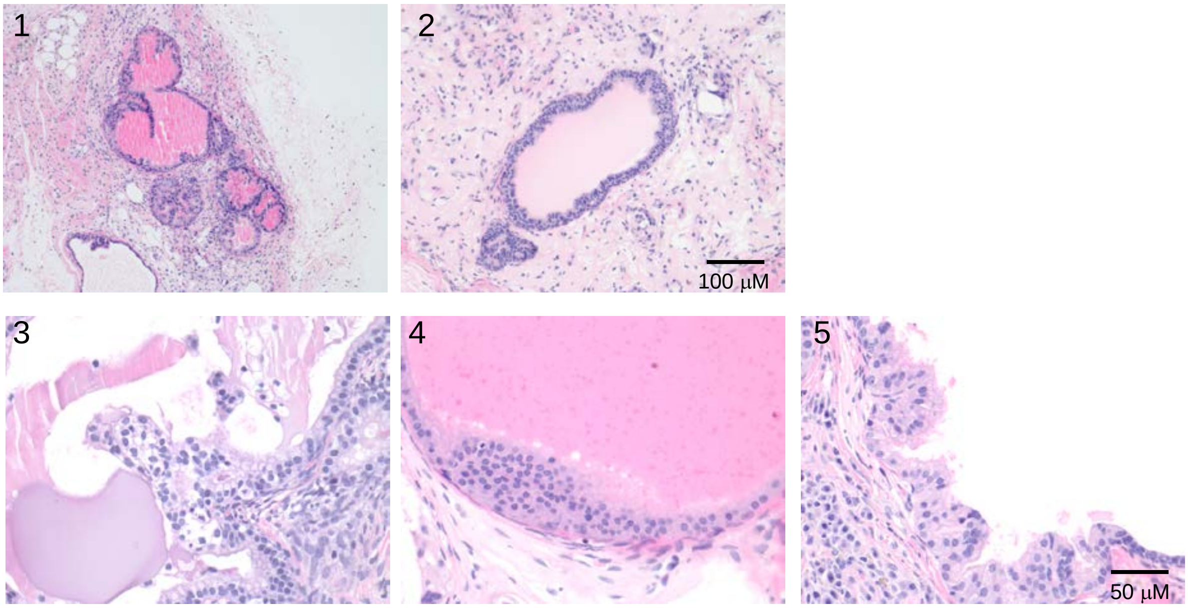

Fig S7

a)

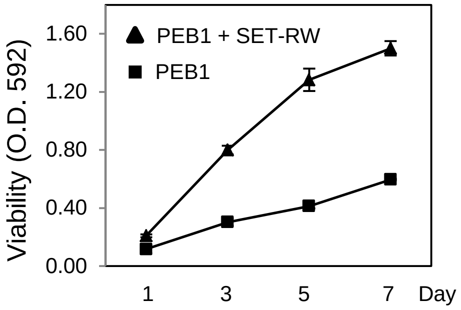
PEB1 + SET-RW
PEB1
Viability (O.D. 592)
1.60
1.20
0.80
0.40
0.00
1
3
5
7
Day

b)

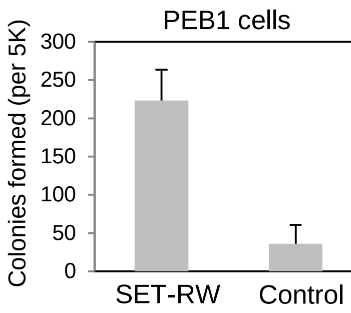
PEB1 cells
Colonies formed (per 5K)
300
250
200
150
100
50
0
SET-RW
Control

Fig S8

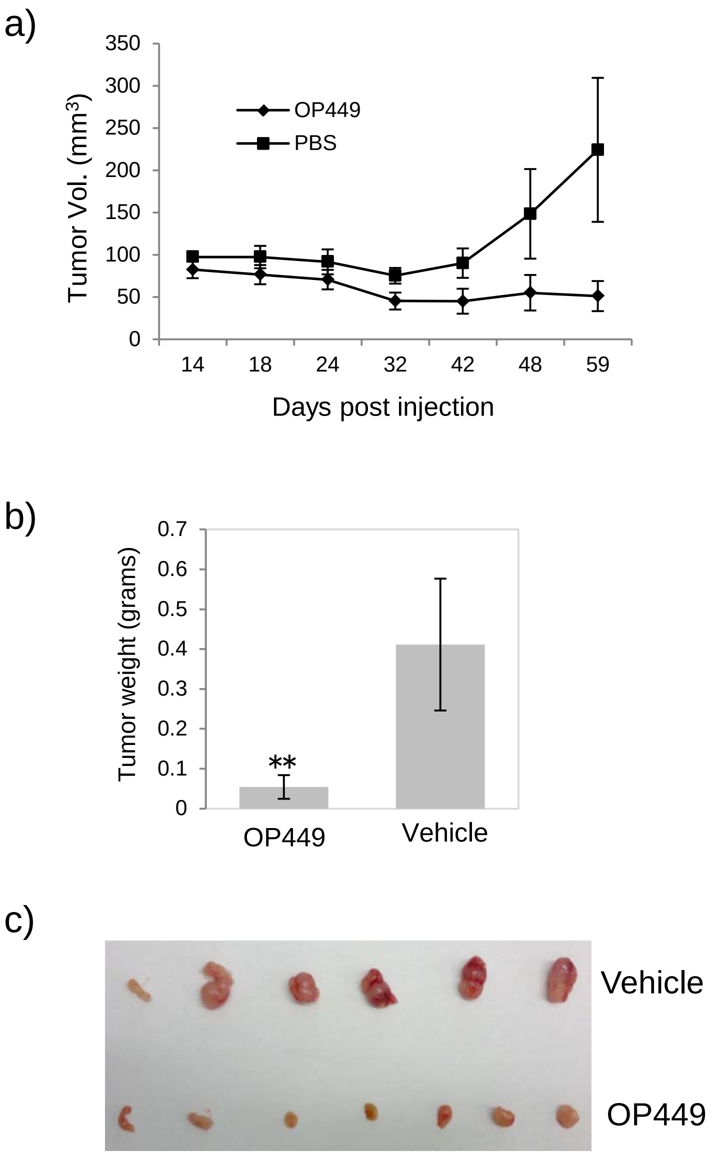

a)
Tumor Vol. (mm^3)
350
300
250
200
150
100
50
0
OP449
PBS
14
18
24
32
42
48
59
Days post injection
b)
Tumor weight (grams)
0.7
0.6
0.5
0.4
0.3
0.2
0.1
0
**
OP449
Vehicle
c)
Vehicle
OP449

Fig S9

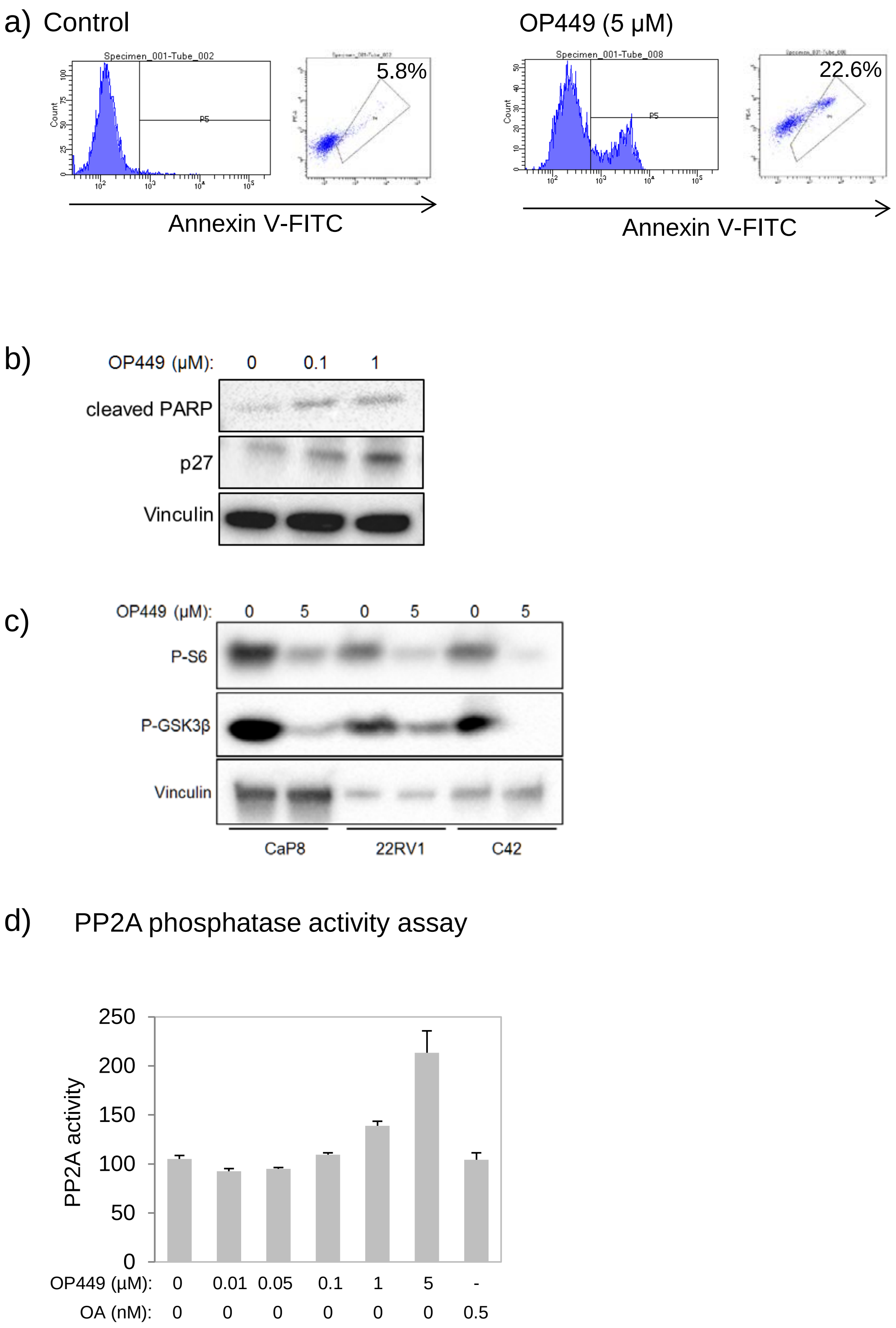
a) Control
Specimen_001-Tube_002
Count
P5
5.8%
Annexin V-FITC
OP449 (5 µM)
Specimen_001-Tube_008
Count
P5
22.6%
Annexin V-FITC
b)
OP449 (µM): 0 0.1 1
cleaved PARP
p27
Vinculin
c)
OP449 (µM): 0 5 0 5 0 5
P-S6
P-GSK3β
Vinculin
CaP8
22RV1
C42
d) PP2A phosphatase activity assay
PP2A activity
250
200
150
100
50
0
OP449 (µM): 0 0.01 0.05 0.1 1 5 -
OA (nM): 0 0 0 0 0 0 0.5

Fig S10

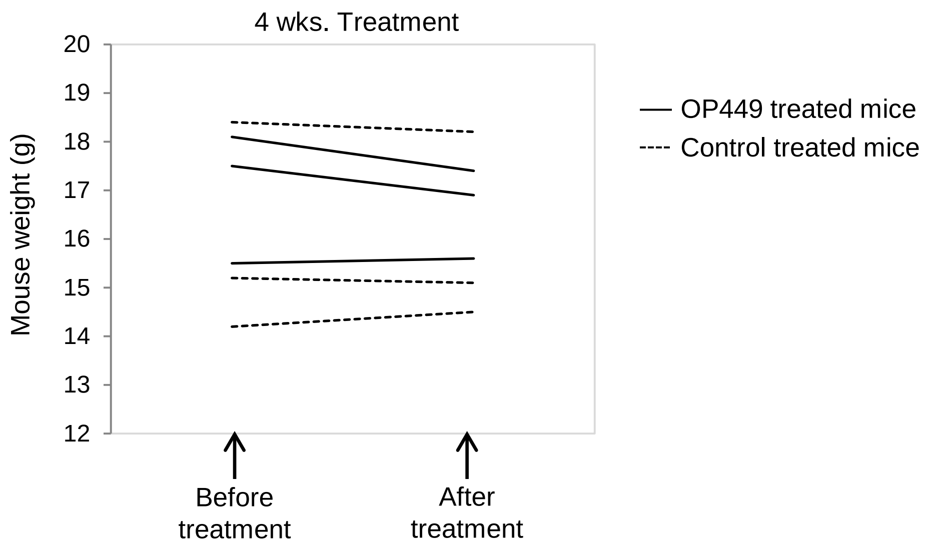

4 wks. Treatment
Mouse weight (g)
20
19
18
17
16
15
14
13
12
Before treatment
After treatment
OP449 treated mice
Control treated mice
